# Effects of grape seed proanthocyanidin B2 pretreatment on oxidative stress and renal tubular epithelial cell apoptosis after renal ischemia reperfusion in mice ^1^

**DOI:** 10.1590/s0102-865020200080000002

**Published:** 2020-09-04

**Authors:** Zhi-shun Wang, Hai-hong Zhou, Qi Han, Yong-lian Guo, Zhong-yuan Li

**Affiliations:** I PhD, Department of Urology, The Central Hospital of Wuhan , Tongji Medical College , Huazhong University of Science and Technology , Wuhan , China . Conception and design of the study, acquisition and analysis of data, manuscript writing.; II PhD, Department of Urology, The Central Hospital of Wuhan , Tongji Medical College , Huazhong University of Science and Technology , Wuhan , China . Conception and design of the study, acquisition and analysis of data.; III Postgraduate, Department of Nephrology , The Fifth Hospital of Wuhan , China . Technical procedures, acquisition and interpretation of data.; IV PhD, Full Professor, Department of Urology, The Central Hospital of Wuhan , Tongji Medical College , Huazhong University of Science and Technology , Wuhan , China . Design of the study, critical revision.; V PhD, Department of Urology, The Central Hospital of Wuhan , Tongji Medical College , Huazhong University of Science and Technology , Wuhan , China . Design of the study, acquisition of data, supervised all phases of the study.

**Keywords:** Grape Seed Extract, Proanthocyanidins, Reperfusion Injury, Oxidative Stress, Apoptosis, Mice

## Abstract

**Purpose:**

To investigate the effects of grape seed proanthocyanidin B2 (GSPB2) preconditioning on oxidative stress and apoptosis of renal tubular epithelial cells in mice after renal ischemia-reperfusion (RIR).

**Methods:**

Forty male ICR mice were randomly divided into 4 groups: Group A: mice were treated with right nephrectomy. Group B: right kidney was resected and the left renal vessel was clamped for 45 minutes. Group C: mice were intraperitoneally injected with GSPB2 before RIR established. Group D: mice were intraperitoneally injected with GSPB2 plus brusatol before RIR established. Creatinine and urea nitrogen of mice were determined. Pathological and morphological changes of kidney were checked. Expressions of Nrf-2, HO-1, cleaved-caspase3 were detected by Western-blot.

**Results:**

Compared to Group B, morphology and pathological damages of renal tissue were less serious in Group C. Western-blot showed that expressions of Nrf-2 and HO-1 in Group C were obviously higher than those in Group B. The expression of cleaved-caspase3 in Group C was significantly lower than that in Group B.

**Conclusion:**

GSPB2 preconditioning could attenuate renal oxidative stress injury and renal tubular epithelial cell apoptosis by up-regulating expressions of Nrf-2 and HO-1 and down-regulating the expression of cleaved-caspase-3, but the protective effect could be reversed by brusatol.

## Introduction

Ischemia-reperfusion Injury (I/R) is a phenomenon in which the injury of tissue and organ is aggravated after the recovery of blood flow based on organ or tissue ischemia. Ischemia-reperfusion injury cannot be avoided in renal transplantation, nephron-sparing surgery and renal parenchymal stone extraction. For a long time, ischemia-reperfusion injury has been a difficult and hot point in clinical treatment. The specific mechanism of renal ischemia-reperfusion injury is still unclear. In recent years, based on the traditional theories of the mechanism of ischemia-reperfusion injury such as calcium overload, oxygen free radicals, inflammatory reaction, necrosis and apoptosis ^1 - 3^ , it was further found that signal pathway, autophagy, new G protein coupled receptor family, G protein subunits, small nucleic acid molecules (miRNA), gas molecules (ammonia gas, ammonia sulfide) and so on could be involved in this process, and it provides a new explanation and intervention target for the mechanism of ischemia-reperfusion injury.

Proanthocyanidins (PC) is a general term for a large class of polyphenols. In recent years, many kinds of proanthocyanidins in various plants, especially in grapes, have been extensively and deeply studied abroad. It has been proved that PC has superior antioxidant activity, enzyme inhibition activity, vascular protection activity, anti-inflammatory activity, anti radiation and anti-tumor activity ^4 - 7^ . Procyanidins are formed by condensation of different amounts of catechins, epicatechins or gallic acid. The simplest procyanidins are dimers formed by catechins and epicatechins, as well as trimers, tetramers and decamers. According to the degree of polymerization, two to four polymers are usually called oligomeric procyanidins. Those above the pentamer are called homopolymers. Oligomeric proanthocyanidins are water-soluble substances, which are easily absorbed. Their ability to scavenge free radicals is related to their molecular structure and degree of polymerization ^8^ . Dimers are the most widely distributed, most antioxidant and most important type of proanthocyanidins ^9^ . In addition, the research shows that grape seed proanthocyanidins B2 has the effects of anti lipid body oxidation, free radical scavenging, anti atherosclerosis, anti-tumor, anti-inflammatory, anti apoptosis and so on, and shows better effect than crude proanthocyanidins extract ^10^ . However, to our knowledge, the effect of proanthocyanidins B2 preconditioning on oxidative stress injury and apoptosis of renal tubular epithelial cells in the mice model of renal ischemia-reperfusion has not been studied until now.

Therefore, this study focused on the effects of GSPB2 preconditioning on oxidative stress injury and apoptosis of renal tubular epithelial cells after renal ischemia reperfusion in mice and its mechanism.

## Methods

The experimental protocol used in this research was approved by the Animal Ethics Review Committee of Huazhong University of Science and Technology, and the entire procedure followed the guidelines of the National Institutes of Health for the care and use of laboratory animals. Forty SPF male ICR mice aged from 6 to 8 weeks were acquired from Experimental Animal Research Center of Tongji Medical College affiliated to Huazhong University of Science and Technology. Animals were housed at this Experimental Animal Research Center based on standard guidelines. The mice were kept in a room with air filtration, a constant temperature (20-22°C), and light control (8 a.m. to 8 p.m.). All animals were free to eat standard food.

### *Experimental protocol*

Mice were anaesthetized by isoflurane inhalation (5% isoflurane initially followed by maintenance on 2.5% with oxygen). Mice were placed on an electric heating pad to maintain constant body temperature at 37°C and performed right nephrectomy. The artery and vein of left kidney were clipped by an atraumatic microvascular clamp, and the clamp was released after 45 min of ischemia, and restoration of blood-flow was confirmed when the kidney regained its normal color. The abdominal wound was sutured with 5/0 suture in two layers, and then 0.5ml PBS was intraperitoneally injected to maintain fluid balance and was kept warm under the heat lamp. The left kidney got reperfusion for 24h. Finally, all mice were killed.

Mice were randomized into four groups: Group A (n=10): mice underwent right nephrectomy but without modeling of the left renal ischemia; Group B (n=10): mice were performed with right nephrectomy and model of left renal ischemia was made for 45 min and then they got reperfusion of 24 h; Group C (n=10): mice were intraperitoneally injected with grape seed proanthocyanidin B2(30 mg/kg) 45 minutes before the ischemia-reperfusion injury model was established, and then followed the same operation as group B; Group D (n=10): mice were intraperitoneally injected with grape seed proanthocyanidin B2 (30 mg/kg) plus Nrf2 inhibitor (Brusatol, Bru, 1 mg/kg) 45 minutes before the ischemia-reperfusion injury model was established, and then followed the same operation as group B.

### *Serum assays*

Twenty-four hours after reperfusion, all the mice were killed to acquire blood samples from the inferior vena cava for testing the concentration of serum creatinine and urea nitrogen. Commercial kits (Nanjing Jiancheng Institute of Biological Engineering, Nanjing, China) were used to detect serum urea nitrogen and creatinine.

### *Histological examination*

The animals were sacrificed after blood sampling; the left kidney was quickly removed, the middle kidney was cut open, the renal capsule was carefully removed, fixed in 10% formaldehyde solution, soaked in 0.01mol/L PBS overnight, dehydrated with conventional gradient alcohol and embedded, and prepared into 4mm paraffin sections. After dewaxing with xylene and rehydration with gradient ethanol, hematoxylin-Eosin (HE) staining was conducted, followed by dehydration with gradient ethanol and xylene, and finally neutral resin was added to seal the tablet.

The pathological and morphological changes of the kidney were assessed by an uninformed professional pathologist. The Olympus BX53F bright field microscope was used to take pictures with the magnification of 400 times. According to the kidney injury scoring method adopted by Jablonski *et al* . ^11^ , proximal renal tubular injury caused by ischemia-reperfusion injury was evaluated, which was divided into five grades from 0 to 4 according to the degree of injury.

### *Periodic acid-Schiff staining*

The sections were dewaxed with conventional water and soaked with distilled water for 1min. A drop (100µl) of 0.5-1% (v/v) periodate acid was added for dyeing for 5~10 min, and distilled water was rinsed for 3 times. One drop (100µl) of Schiff’s solution was added for staining for 10~30 min and Schiff’s solution was poured. One drop (100µl) of sodium bisulfite was added directly for 2 minutes, 2 times in total. It was rinsed with running water for 10 min to make it appear red. It was washed once with distilled water. The nuclei were restained with hematoxylin solution for 2min. Conventional dehydration, transparent, neutral gum sealing.

### *Terminal deoxynucleotidyl transferase dUTP nick end labeling (TUNEL) assay*

Apoptosis in renal tissue induced by ischemia-reperfusion injury was determined using the in situ apoptosis detection kit (Promega Corpora,USA). The whole test was carried out according to the kit instructions. Specifically, the slices were immersed overnight in a 4% paraformaldehyde/phosphate buffer (PBS) with a PH of 7.4 at 4°C. Then, the slices were rinsed with PBS (1×) solution for 3 consecutive times, and then soaked in 70% ethanol for 24 h at a temperature of 20°C. The slices were then washed three times with PBS (1×), and then soaked in permeable buffer on the ice for 15 minutes, and then washed with PBS (1×). The sections were then incubated with 50 mL reaction buffer (5 mL terminal deoxidized nucleotide transferase and 45 ml labeled safety buffer) (Promega Corporation) for 90 minutes at 37˚C. Finally, the slices were washed with PBS (1×) to terminate the marking process. The images were captured and analyzed using confocal laser scanning microscopy (Carl Zeiss AG, Germany). The average number of apoptotic cells per 100 cells was counted from 5 high-power microscope fields randomly selected from the distribution area of apoptotic cells in each slice. Apoptosis index (AI) was calculated as a percentage.

### *Measurement of SOD, MDA, CAT and GSH levels in kidney*

SOD activity in kidney of mice was measured by xanthine oxidase assay. The absorbance at 550 nm was determined by spectrophotometer. The concentration of MDA in renal tissues of mice in each group was determined by thiobarbituric acid method, and the level of MDA was reflected by the level of lipid peroxides. The absorbance value at 532 nm was determined by spectrophotometer. The activity of CAT in renal tissues of mice in each group was measured by visible light using the detection kit. Spectrophotometric determination of absorbance was performed at 405 nm. The content of GSH in renal tissues of mice in each group was determined by colorimetry using the detection kit. The absorbance at 412 nm was determined by spectrophotometer. The above SOD, MDA, CAT and GSH detection kits were purchased from Nanjing Jieching Biological Engineering Research Institute, and coomassie blue method was used to determine the homogenate protein concentration of mouse kidney tissues in each group.

### *Immunohistochemistry*

Immunohistochemical staining was used to detect Nrf2, HO-1 and Cleaved Caspase-3 protein expressions in renal tissues of mice in each group. Specifically, the sections were dewaxed to water, and the endogenous peroxidase was blocked at 37°C with 3% hydrogen peroxide for 10 minutes. The treated tissue sections were placed in the prepared EDTA antigen repair solution for antigen repair, and then the sections were sealed at 37°C for 30 minutes with 10% normal goat serum in the kit. Then the primary antibodies were added: rabbit anti-mouse Nrf-2 polyclonal antibody (Nrf2; Dilution at 1:300; Santa Cruz Biotechnology, Santa Cruz, CA); Rabbit anti-mouse HO-1 polyclonal antibody (HO-1; Dilution at 1:1000; Wuhan Sanying Biotechnology, Wuhan, China); Rabbit anti-mouse Cleaved caspase-3 Polyclonal antibody (Cleaved caspase-3; dilution at 1:1000; Cell Signaling Technology, Boston, USA). All of them were incubated overnight at 4°C. Then, sections were incubated with secondary anti-rabbit antibody IgG labeled with Horseradish peroxidase (1:5000, Boster, Wuhan, China) for half an hour at room temperature, and coloured by DAB. For the negative control group, phosphate buffer was used instead of primary antibody as negative control.

### *Western Blot analysis*

Tissue proteins were extracted and purified from mouse kidneys as before ^12^ . Specifically, each group of protein samples was subjected to gel electrophoresis with 12.5% sodium dodecyl sulfate-polyacrylamide gel (40µg/Lane) for protein separation, and the proteins were then electrotransferred to nitrocellulocellulose membrane (Bio-RAD). The membrane was then sealed with TBST buffer containing 5% skimmed milk powder. Then primary antibodies were added and incubated under 4˚C environment overnight. The membrane was washed three times on a shaker with TBST buffer solution, then secondary antibodies were added, the membrane was incubated, and then the enhanced chemical brighteners eECL-A and eECL-B (ECL kit;Pierce Biotechnology, Rockford, IL) were mixed in the same proportion and volume and added to the membrane evenly, exposure, analysis with photosensitive imaging film (Kodak). Primary antibodies used in this study include Rabbit anti-Mouse Nrf-2 polyclonal antibody (Nrf2; Dilution at 1:300; Santa Cruz Biotechnology, Santa Cruz, CA); Rabbit anti-mouse HO-1 polyclonal antibody (HO-1; Dilution at 1:1000; Wuhan Sanying Biotechnology, Wuhan, China); Rabbit anti-mouse Cleaved Caspase-3 polyclonal antibody (Cleaved Caspase-3; Dilution at 1:1000; Cell Signaling Technology, Boston, USA). An anti-rabbit IgG antibody labelled with horseradish peroxidase with a secondary antibody (Boster, Wuhan, China). GAPDH was used as internal reference to ensure the same amount of protein in each lane. Western blot results were analyzed using Image J software. We quantified the results by calculating the average gray value of each protein.

### *Statistical analysis*

SPSS statistical software (Version 18.0) was used to analyze all experimental data, and Student’s T-test was used to test the difference between the mean values of different groups. When P˂0.05, the difference was considered statistically significant.

## Results

### *Effect of grape seed proanthocyanidin B2 (GSPB2) preconditioning on renal function after renal ischemia-reperfusion*

**Figure 1:**
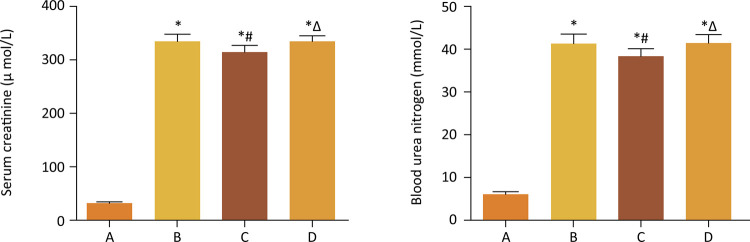
Effects of GSPB2 preconditioning on the renal function after renal ischemia reperfusion in mice model. Both serum Cr and BUN concentrations in groups B, C and D were obviously higher than those in group A. Furthermore, the levels of serum Cr and BUN in group C were significantly lower than those in group B, and there were significant differences in the levels of serum Cr and BUN between groups B and C. However, brusatol could reverse the protective effect of GSPB2 pretreatment on renal function after renal ischemia-reperfusion injury in group D, and there were significant differences in the levels of serum Cr and BUN between groups C and D.*p<0.05 *vs* . group A. # p<0.05 *vs* . group B. Δ p<0.05 *vs* . group C.

Renal function of mice was measured 24 h after renal ischemia-reperfusion injury. Serum urea nitrogen and serum creatinine levels were obviously increased in the renal ischemia-reperfusion group compared with the sham operated group. Besides, renal function damage caused by ischemia-reperfusion injury could be significantly improved by GSPB2 preconditioning. However, brusatol reversed the protective effect of GSPB2 pretreatment on renal function after renal ischemia-reperfusion injury in mice (Fig. 1).

### *Effect of GSPB2 preconditioning on morphological lesions after renal ischemia-reperfusion*

**Figure 2:**
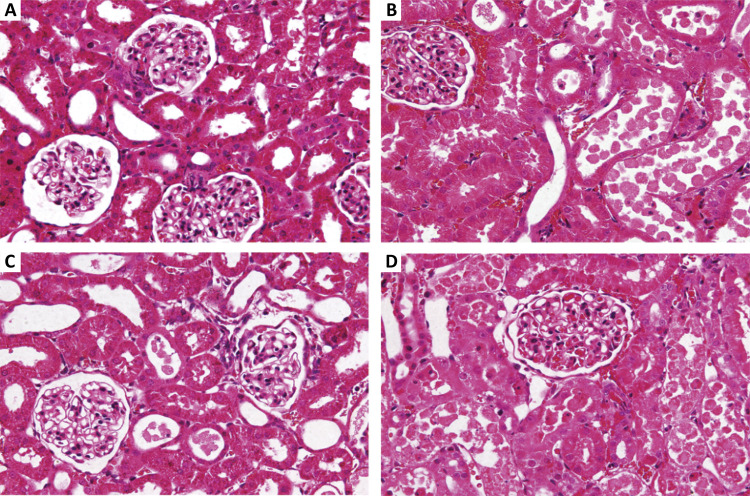
GSPB2 preconditioning alleviated the morphological damages after renal ischemia-reperfusion according to the representative micrographs of hematoxylin-eosin staining (×400). There were serious morphological lesions in group B, including renal tubular swelling, necrosis and the destruction of renal tubular normal construction. Furthermore, compared to group B, morphological damages in group C were obviously attenuated. However, the protective effect of GSPB2 pretreatment on renal ischemia-reperfusion injury in mice could be reversed by brusatol in group D.

**Figure 3:**
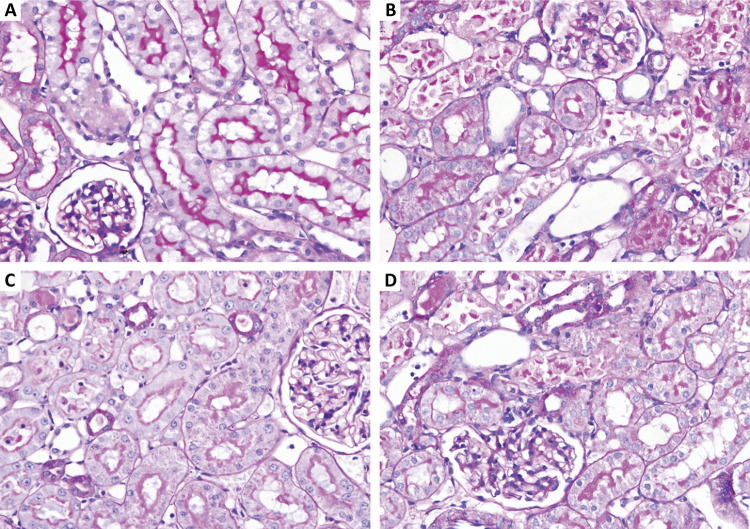
GSPB2 preconditioning attenuated the injury of brush border of proximal renal tubular after renal ischemia-reperfusion. Representative micrographs of PAS staining showed that severe renal damages including tubular necrosis and destruction of proximal renal tubular brush border were obviously attenuated in group C than those in group B (×400). However, The protective effect of GSPB2 pretreatment on renal ischemia-reperfusion injury in mice could be reversed by brusatol in group D.

**Figure 4:**
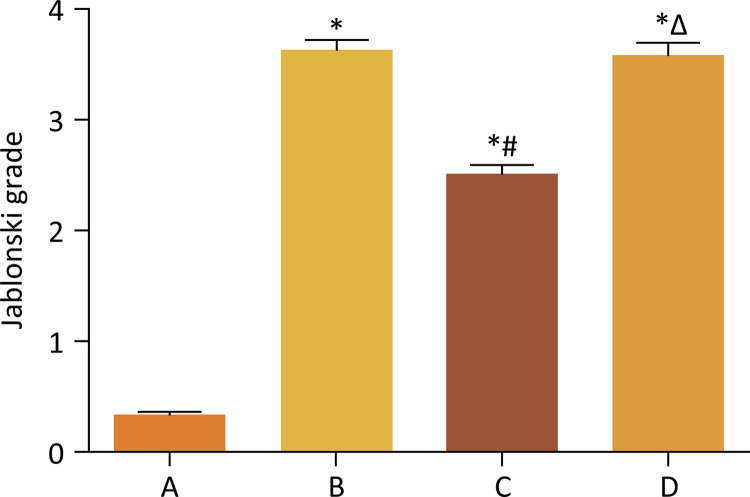
GSPB2 preconditioning reduced the Jablonski grade after renal ischemia-reperfusion. The Jablonski grades in groups B, C and D were obviously higher than those in group A. Besides, Jablonski grade in group C was significantly lower than that in group B. Meanwhile, Jablonski grade in group D was obviously higher than that in group C. *p<0.05 *vs* . group A, # p<0.05 *vs* . group B, Δ p<0.05 *vs* . group C.

**Figure 5:**
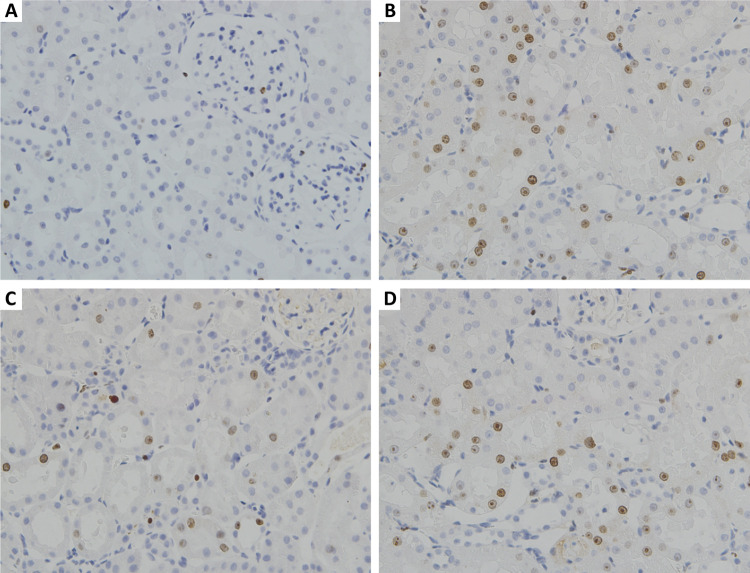
Effects of GSPB2 pretreatment on apoptosis of renal tubular epithelial cells induced by ischemia-reperfusion injury. TUNEL assay was used to detect apoptosis of renal tubular epithelial cells (×400).

Histopathological examination showed that there was pathological damage and morphological changes in the kidney of mice after renal ischemia-reperfusion injury. However, GSPB2 preconditioning could alleviate the extent of renal morphology damages. Meanwhile, brusatol could reverse the protective effect of GSPB2 pretreatment after renal ischemia-reperfusion injury. Renal ischemia-reperfusion injury could lead to significant renal damage in many aspects, including tubules epithelial swelling, necrosis, tubules dilatation, and brush edge loss. However, these renal damages could be alleviated by GSPB2 preconditioning (Figs. 2 and 3). Besides, there were less renal tubular necrotizing changes in the group C than those in group B. The Jablonski grade was used to quantitatively analyze the severity of acute tubular injury, and it was found that the Jablonski grade in group C was significantly lower than that in group B (Fig. 4). However, these renal damages and Jablonski grade were worse in group D than those in group C (Figs. 2 to 4). In addition, GSPB2 preconditioning significantly reduced the apoptosis of renal tubular epithelial cell in the group C compared with group B (Fig. 5).

### *Effect of GSPB2 preconditioning on oxidative stress injury after renal ischemia-reperfusion*

As listed in Table 1 , MDA content in kidney tissues of group B, C and D was significantly higher than that of group A. Furthermore, the content of MDA in the kidney tissues of group C was significantly lower than that of group B. However, MDA content in kidney tissues of group D was obviously higher than that of group C. Meanwhile, compared with group A, SOD, CAT and GSH levels in kidney tissues of group B, C and D were all significantly reduced. Besides, compared to group B, the reduction of the content of SOD, CAT, GSH after renal ischemia-reperfusion could be obviously attenuated in group C. However, the levels of SOD, CAT, GSH in group D were significantly lower than those in group C.

**Table 1:** Effect of GSPB2 preconditioning on the renal content of the SOD, MDA, CAT, GSH at 24h after renal ischemia reperfusion (n=10).

Groups	SOD(units/mgprot)	MDA(nmol/mgprot)	CAT(U/mgprot)	GSH(µmol/gprot)
A	84.79±6.98	3.48±1.26	73.13±5.78	40.62±5.06
B	39.34±7.95*	7.14±1.59*	33.97±6.12*	13.18±3.59*
C	54.96±8.87* ^,#^	4.89±1.45* ^,#^	56.32±6.93* ^,#^	28.38±5.34* ^,#^
D	40.98±9.76* ^,Δ^	7.06±1.78* ^,Δ^	34.17±6.48* ^,Δ^	14.25±4.81* ^,Δ^

The experimental results were mean±standard error. *P<0.05 *vs* . group A, #P<0.05 *vs* . group B. ΔP<0.05 *vs* . group C.

SOD, superoxide dismutase; MDA, malondialdehyde; CAT, Catalase; GSH, glutathione.

### *Effect of GSPB2 preconditioning on the expression levels of Nrf2, HO-1 and Cleaved-Caspase3 after renal ischemia-reperfusion*

**Figure 6:**
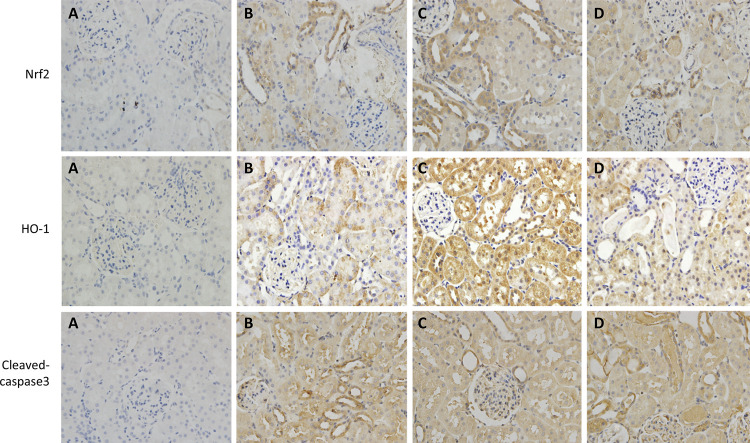
GSPB2 pretreatment could significantly increase the protein expression levels of Nrf2 and HO-1 in the renal tissue of mice after ischemia reperfusion injury according to the immunohistochemical representative images (×400). Meanwhile, GSPB2 pretreatment could also restrain the expression level of Cleaved-Caspase3 in renal tissue after renal ischemia-reperfusion according to the representative micrographs of immunohistochemistry (×400). Compared to group B, protein expression levels of Nrf2 and HO-1 in kidney tissues of mice in group C were significantly increased. Furthermore, the expression level of Cleaved-Caspase3 in group C was obviously lower than that in group B. However, compared to group C, protein expression levels of Nrf2 and HO-1 in kidney tissues of mice in group D were significantly reduced. In addition, the expression levels of Cleaved-caspase3 in group D were obviously higher than those in group C.

**Figure 7:**
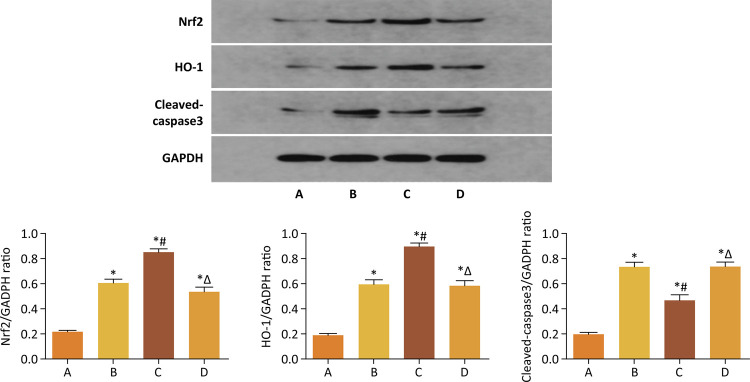
GSPB2 pretreatment could significantly increase the protein expression levels of Nrf2 and HO-1 in the renal tissue of mice after ischemia reperfusion injury according to the results of western blot. Meanwhile, GSPB2 preconditioning also restrained the expression level of Cleaved-Caspase3 in renal tissue after renal ischemia-reperfusion. Compared to group B, protein expression levels of Nrf2 and HO-1 in kidney tissues of mice in group C were significantly increased. Furthermore, the expression level of Cleaved-Caspase3 in group C was obviously lower than that in group B. However, compared to group C, protein expression levels of Nrf2 and HO-1 in kidney tissues of mice in group D were significantly reduced. In addition, the expression levels of Cleaved-caspase3 in group D were obviously higher than those in group C.*p<0.05 *vs* . group A, #p<0.05 *vs* . group B, Δp<0.05 *vs* . group C.

The results of immunohistochemistry and western blot in this study showed that the protein expression levels of Nrf2 and HO-1 in group C were significantly higher than those in group B. Furthermore, the expression level of Cleaved-caspase3 in group C were significantly lower than that in group B. However, the protein expression levels of Nrf2 and HO-1 in group D were obviously lower than those in group C. In addition, the expression level of Cleaved-caspase3 in group D were obviously higher than that in group C (p<0.05) (Figs. 6 and 7).

## Discussion

As an important organ of human body, the kidney has abundant blood flow and is the organ with high blood perfusion in the body. It is sensitive to renal ischemia, and reperfusion injury can lead to renal microvascular injury, vasoconstriction, reduction of blood supply to the kidney, and eventually cause a sharp decline in renal function ^13 - 15^ . Renal ischemia-reperfusion injury is inevitable during kidney transplantation, nephron-sparing surgery, cardiac arrest and hypotensive shock ^16 , 17^ . In recent years, with the development of kidney transplantation and major heart surgery, the incidence and mortality rate of renal ischemia-reperfusion injury also increased year by year, accounting for the mortality rate 20 percent of hospitalized patients ^18^ . Therefore, in-depth study of the pathophysiological changes and related mechanisms of renal ischemia-reperfusion injury is of great significance for early intervention or prevention measures.

The pathogenesis of renal ischemia-reperfusion injury is complicated, which is a series of processes mediated by various factors and pathways. At present, studies on renal ischemia-reperfusion injury mainly focus on Ca ^2^ overload, cell apoptosis, activation of inflammatory response mechanism, oxygen free radical increase and other areas related to oxidative stress and immune response ^19 - 21^ . At present, in addition to shortening the time of ischemia reperfusion and local low temperature ice compress, studies on protective measures against renal ischemia reperfusion injury also include: ischemic preconditioning, ischemic postconditioning, stem cell transplantation, and new drug therapy ^22 - 25^ . However, recent studies by Arantes *et al* . ^26^ showed that both ischemic preconditioning and post-ischemic treatment, whether alone or in combination, could not contribute to the protection of renal function and could not play a protective role in tubular cell injury after acute renal ischemia-reperfusion injury. Though the treatment of renal ischemia-reperfusion injury by stem cell transplantation has made great progress in animal experiments ^27 , 28^ , there are still few studies in clinical trials, so further verification is still needed.

A large number of studies have reported that grape seed procyanidins have a variety of biological activities, such as anti-oxidative stress, reducing inflammatory response and cell apoptosis, and preventing tumor ^4 - 7^ . In this study, the model of renal ischemia reperfusion injury in mice was established and pretreated with grape seed procyanidin B2, which is the most antioxidant dimer among the oligomers of grape seed procyanidin ^9^ . The experimental results showed that the preconditioning of grape seed proanthocyanidin B2 could significantly improve the activity of SOD, CAT and GSH enzymes in renal tissues damaged by ischemia-reperfusion in mice, and significantly reduce the level of MDA. Through systematic review and meta-analysis, Li *et al* . ^29^ concluded that proanthocyanidin intervention could improve the antioxidant index level of SOD, CAT, GSH, GPx and T-AOC, and reduce the concentration of MDA in the mouse oxidative damage model, among which the dipolymer of proanthocyanidin was the most widely studied and most valued. Zhang *et al* . ^30^ also showed that grape seed proanthocyanidin B2 could significantly reduce oxidative stress injury and apoptosis of porcine ovarian granulosa cells induced by hydrogen peroxide. Pretreatment with grape seed proanthocyanidin B2 could significantly improve the levels of T-AOC, SOD, and GSH-Px in ovarian tissue, reduce the levels of MDA and ROS in ovarian tissue, and significantly up-regulate the over-expression of let-7a gene ^30^ . Studies have shown that the consumption of rich phenolic compounds containing grape seed procyanidin B2 could significantly reduce the urine redox potential, and comprehensively improve the antioxidant capacity ^4^ . Therefore, we speculated that grape seed anthocyanin B2 preconditioning could reduce the oxidative stress injury of kidney damaged by ischemia-reperfusion injury in mice, which may be related to the improvement of the overall antioxidant capacity of mice.

As a basic leucine zipper redox sensitive transcription factor, Nrf2 regulates the expression of many cell antioxidant and cytoprotective genes ^31^ . The Nrf2/HO-1 signaling pathway is an important endogenous antioxidant system in vivo, which plays a crucial role in maintaining the oxidative balance of the body. Among them, Nrf2 is an important transcription factor of endogenous antioxidant stress and is regulated by kelch-like ECH related protein 1 (Keap1). Under normal conditions, Keap1 is an adaptor protein of cullin3 (Cul3)-ring-box 1 (Rbx1) containing the E3 ubiquitin ligase complex that targets Nrf2 for proteasomal degradation to maintain low levels of intracellular Nrf2 protein and blocks transcription of downstream target genes ^32^ . When body cells were exposed to ROS or electron philophiles those were beyond the detoxification function of the body, Keap-1 could activate Nrf-2 by altering its cysteine residues to alter the conformation of Keap-1.The activated Nrf-2 heterodimer was then transferred into the nucleus to bind to the antioxidant or electrophilic reactive element (EpRE) in the Nrf2 target gene promoter region ^33^ . Nrf2 regulated a variety of antioxidant activity genes including heme oxygenase 1 (HO-1), peroxidase (PRDX1), glutathione peroxidase 1 (GPX1), glutamate-cysteine ligase modified subunit (GCLM), NADPH quinine oxidoreductase 1 (NQO1), superoxide dismutase (SOD), catalase (CAT), and so on ^32 , 34^ . In our study, compared with group B, the protein expression levels of Nrf2 and HO-1 in group C were significantly increased. However, after the intervention with brucopicrin (Nrf2 inhibitor), Nrf2 protein and HO-1 protein expression levels in group D were significantly lower than those in group C. The experimental results showed that grape seed proanthocyanidin B2 might improve the expression level of antioxidant stress protein by activating the Nrf2/ HO-1 signaling pathway, and finally alleviate the oxidative stress injury and related pathological changes caused by ischemia reperfusion injury in the kidney of mice. In fact, studies have shown that Nrf2 regulates many cell activities. In addition to direct homeostasis and cell protection, Nrf2 also affects various processes such as inflammation, proliferation, apoptosis, cell differentiation, tissue regeneration and even metabolism ^33^ .

In renal ischemia-reperfusion injury, oxidative stress injury, calcium ion overload, excessive generation of ROS and other free radicals are inevitable, which leads to mitochondrial damage and impaired function, which can promote cell death through activation of apoptosis or necrosis pathway ^35^ . The experimental results showed that the grape seed procyanidin B2 pretreatment could significantly reduce the apoptosis of renal tubular epithelial cells in mice and necrosis, and could significantly reduce the cleaved-caspase3 protein expression level; those might be related to grape seed procyanidin B2 pretreatment to enhance the overall antioxidant system in mice, at the same time reduce the ischemia-reperfusion kidney partial oxidative stress injury. Li *et al* . ^36^ also showed that the intervention of procyanidins B2 could significantly reduce the apoptosis of ARPE-19 cells induced by photooxidation, increase the proportion of bcl-2/Bax in mitochondria, reduce the release of ROS and cytochrome c, and reduce the caspase cleavage. Li *et al* . ^37^ also found that the antioxidant activity of grape seed proanthocyanidin B2 (GSPB2) and resveratrol inhibited the activation of cytochrome c release, caspase-9 and caspase-3 through the regulation of lactadherin, and had a protective effect on advanced glycation end products induced apoptosis.

## Conclusions

This study showed that pretreatment with proanthocyanidin B2 of grape seed could significantly alleviate oxidative stress injury and apoptosis of renal tubular epithelial cells in mice caused by renal ischemia-reperfusion injury, and that this protective effect might be related to the activation of Nrf2/HO-1 signaling pathway. In addition, inhibiting the activation of apoptosis or necrosis pathway caused by oxidative stress may be another reason why pretreatment of grape seed proanthocyanidin B2 plays a protective role. However, the results are limited to mice, and further studies in mammals or clinical trials are needed.
